# Mental distress of physicians in the outpatient care throughout the COVID-19 pandemic: emotional and supportive human relations matter – Cross-sectional results of the VOICE-study

**DOI:** 10.1186/s12913-023-09361-3

**Published:** 2023-05-12

**Authors:** Sabine Mogwitz, Christian Albus, Petra Beschoner, Yesim Erim, Franziska Geiser, Lucia Jerg-Bretzke, Eva Morawa, Susann Steudte-Schmiedgen, Gloria-Beatrice Wintermann, Kerstin Weidner

**Affiliations:** 1 Department of Psychotherapy and Psychosomatic Medicine, Faculty of Medicine, Technische Universitaet Dresden, Dresden, Germany; 2 Department of Psychosomatics and Psychotherapy, Medical Faculty and University Hospital of Cologne, Kerpener Str. 62, Cologne, 50937 Germany; 3 Department of Psychosomatic Medicine and Psychotherapy, Ulm University Medical Center, Albert-Einstein-Allee 23, 89081 Ulm, Germany; 4 Department of Psychosomatic Medicine and Psychotherapy, University Hospital of Erlangen, Friedrich-Alexander University Erlangen-Nürnberg (FAU), Maximiliansplatz 2, 91054 Erlangen, Germany; 5 Department of Psychosomatic Medicine and Psychotherapy, University Clinic of Bonn, Venusberg-Campus 1, 53127 Bonn, Germany

**Keywords:** COVID-19, Physicians, General practioner, Outpatient/ inpatient care, Mental distress, COVID-19-related, Work-related risks/ resources, Social support, Optimism

## Abstract

**Background:**

The aim of this cross-sectional study was to evaluate the course of self-reported mental distress and quality of life (QoL) of physicians, working in the outpatient care (POC). Outcomes were compared with a control group of physicians working in the inpatient care (PIC), throughout the Corona Virus Disease (COVID)-19 pandemic. The impact of risk and protective factors in terms of emotional and supportive human relations on mental distress and perceived QoL of POC were of primary interest.

**Methods:**

Within the largest prospective, multi-center survey on mental health of health care workers (HCW), conducted during the first (T1) and second (T2) wave of the COVID-19 pandemic in Europe, we investigated the course of current burden (CB), depression (Patient Health Questionnaire-2), anxiety (Generalized Anxiety Disorder-2) and QoL, cross-sectionally, in *n* = 848 POC (T1: *n* = 536, T2: *n* = 312). The primary outcomes were compared with an age- and gender-matchted control group of *n* = 458 PIC (T1: *n* = 262, T2: *n* = 196). COVID-19-, work-related, social risk and protective factors were examined.

**Results:**

At T1, POC showed no significant differences with respect to CB, depression, anxiety, and QoL, after Bonferroni correction. Whereas at T2, POC exhibited higher scores of CB (Cohen´s d/ Cd = .934, *p* < .001), depression (Cd = 1.648, *p* < 001), anxiety (Cd = 1.745, *p* < .001), work-family conflict (Cd = 4.170, *p* < .001) and lower QoL (Cd = .891, p = .002) compared with PIC. Nearly all assessed parameters of burden increased from T1 to T2 within the cohort of POC (e.g. depression: CD = 1.580, *p* < .001). Risk factors for mental distress of POC throughout the pandemic were: increased work-family conflict (CB: ß = .254, *p* < .001, 95% CI: .23, .28; PHQ-2: ß = .139, *p* = .011, 95% CI: .09, .19; GAD-2: ß = .207, *p* < .001, 95% CI: .16, .26), worrying about the patients´ security (CB: ß = .144, p = .007, 95% CI: .07, .22; PHQ-2: ß = .150, p = .006, 95% CI: .00, .30), fear of triage situations (GAD-2: ß = .132, p = .010, 95% CI: -.04, .31) and burden through restricted social contact in spare time (CB: ß = .146, p = .003, 95% CI: .07, .22; PHQ-2: ß = .187, *p* < .001, 95% CI: .03, .34; GAD-2: ß = .156, p = .003, 95% CI: -.01, .32). Protective factors for mental distress and QoL were the perceived protection by local authorities (CB: ß = -.302, *p* < .001, 95% CI: -.39, -.22; PHQ-2: ß = -.190, *p* < . 001, 95% CI: -.36, -.02; GAD-2: ß = -.211, *p* < .001, 95% CI: -.40, -.03; QoL: ß = .273, *p* < .001, 95% CI: .18, .36), trust in colleagues (PHQ-2: ß = -.181, *p* < .001, 95% CI: -.34, -.02; GAD-2: ß = -.199, *p* < .001, 95% CI: -.37, -.02; QoL: ß = .124, p = .017, 95% CI: .04, .21) and social support (PHQ-2: ß = -.180, *p* < .001, 95% CI: -.22, -.14; GAD-2: ß = -.127, p = .014, 95% CI: -.17, -.08; QoL: ß = .211, *p* < .001, 95% CI: .19, .23).

**Conclusions:**

During the pandemic, the protective role of emotional and supportive human relations on the mental distress and quality of life of POC should be taken into account more thoroughly, both in practice and future research.

**Supplementary Information:**

The online version contains supplementary material available at 10.1186/s12913-023-09361-3.

## Background

An increasing body of research revealed the various consequences of the Corona Virus Disease (COVID)-19 pandemic for patient care in all medical fields [1–4]. The resulting psychosocial burden and mental distress for health care workers (HCW), working in clinics during the COVID-19 pandemic, has been well investigated [5–9]. However, drawing general conclusions remains difficult e.g. due to different strategies in handling the COVID-19 pandemic internationally. Of note, mainly inpatient care has been focused on so far [10]. Current evidence showed an increased risk for depression, anxiety and burnout in HCW treating infected patients in clinics [5, 11, 12]. In fact, several work- and COVID-related risk factors for mental distress in clinical HCW could be identified, e.g. when the intensive care for patients with severe COVID-19 had to be expanded; elective surgeries or other medical interventions had to be postponed, leading to an increased strain on clinical HCW [13, 14]. Furthermore, patients themselves cancelled appointments, examinations and surgeries due to the fear of contagion [10]. Based on this situation, HCW anticipated concerns of collateral damage to the health of the population due to abandoned or postponed routine care [15–17].

However, during the acute situation of the pandemic, an increased demand for outpatient care, arised as well [10]. General practitioners (GPs) or physicians working in the outpatient care (POC) stood in the frontline throughout the COVID-19 pandemic [18]. In fact, 85% of patients with COVID-19 were treated by GPs [18, 19]. Moreover, nearly 94% of polymerase chain reaction (PCR) tests for suspected COVID-19 infections were provided in the outpatient care during the first quarter of 2020 in Germany. Additionally, long-term sequelae of COVID-19 necessitated a prolonged post-discharge medical monitoring and outpatient treatment (for a systematic review see [20]). Overall, GPs contributed substantially to the prevention of an overload of hospitals [10, 21]. Consequently, GPs are essential for providing and maintaining health care during a pandemic, since they play a key role in each phase of response to the virus by monitoring and treating patients.

However, working in the frontline with suspected COVID-19 infected patients, GPs are frequently exposed to the virus themselves, making them a potential source of community spread, if not adequately protected by the appropriate personal protective equipment (PPE) [22]. Early on during the pandemic, difficulties in finding PPE arose, and accurate information on sufficient protection was missing [19]. In consequence, GPs had called for the same appropriate PPE in the outpatient care as in the inpatient care [23]. Recently, the direct impact of the lack of preparedness on outpatient care was highlighted and consequently pandemic preparedness of GPs was anticipated to play a pivotal role in the future management of the pandemic situation [19, 24–30]. Of note, lack of PPE, training and information access as well as support from authorities may contribute to emotional burden and worries in every day work [19, 31, 32]. In line, results confirm that work safety aspects are inversely associated with the risk of burnout in medical staff during the COVID-19 pandemic [12].

Furthermore, uncertainty about the pandemic progression, changes in practice organization, increased workload, social tension within teams, worries and fears of infection may have an impact on the mental and physical well-being of GPs [10]. Some of these factors also seem to have negatively influenced the work climate in the outpatient care, since more than one fifth of GPs reported a worsening of the work climate during the COVID-19 pandemic. This was assessed to be more burdensome than the practice staff’s fears about the risk of transmitting the virus [33]. In fact, former research revealed an association between dissatisfaction with work climate and an increased risk of mental health disorders and burnout [34]. From this point of view, aspects of internal communication seem to have a higher potential, regarding the impact on work climate, than individual fears or lack of protective materials [33].

Moreover, colleagues and patients in need could not be appropriately supported due to the restrictive requirements of infection control such as isolation when infected [35]. Interestingly, research has indicated that GPs worry about infecting others or burdening colleagues by not contributing their part of work. The mentioned aspects turned out to be of greater concern than becoming ill themselves [19]. In line, results of previous research suggest that interindividual factors such as social support, but also individual resources such as optimism and sense of coherence may have an important, protective impact on the mental well-being in HCW [7, 36–38].

To sum up, facing the COVID-19 pandemic, GPs are daily exposed to several physical and psychological challenges [11] but literature on the burden and mental distress of physicians in the outpatient care remain scarce. However, only a few studies have given insight into mental distress and quality of life in physicians in the outpatient care (POC) during infectious disease public health crises [10, 19, 32, 33, 39]. Along with associated risk and protective factors, aspects of emotional human and supportive relations among POC and stratification of work-settings throughout the pandemic have not been investigated sufficiently, so far. POC usually do not belong to teams as large as in clinical settings and their moral concerns are based on often long-term, close relationship to their patients. Although POC and physicians working in the inpatient care (PIC) share great responsibilities for their patients, both remain quite heterogenous groups. Following, one might assume that specific aspects of mental health of POC and its course differ from PIC, throughout the pandemic. The impact of aspects involving emotional and supportive relations in private and work-life on mental distress in POC during the COVID-19 pandemic has not been in the focus so far.

Therefore, the present investigation aims to contribute to the elucidation of risk and protective factors of mental distress such as sociodemographic, work- and COVID-19-related factors in POC throughout the pandemic. We hypothesize a significant psychosocial burden in POC along with less perceived support e.g. through colleagues, employers, local authorities and family, compared to their colleagues in the inpatient care (PIC), at the beginning and throughout the first year of the COVID-19 pandemic. We further suppose existing intraindividual and interindividual contributors, such as work- and covid-related variables to be influential on mental distress and perceived quality of life, throughout the pandemic. Emotional and supportive human relations between physicians and patients, colleagues, authorities or relatives such as perceived support and protection, may contribute significantly to mental distress and perceived quality of life. Interpersonal aspects may demonstrate a stronger association than sociodemographic and individual aspects such as gender, age, family status or being at risk for a severe COVID-19-infection.

## Methods

### Participants and procedures

The present study results are part of the prospective study “VOICE,” conducted within the framework of the egePan Unimed project ‘Development, testing and implementation of regionally adaptive care structures and processes for evidence-based pandemic management coordinated by university medicine’ [7]. The online survey aimed to assess mental health, stressors and resources of HCW during the COVID-19 pandemic. It was conducted throughout the first to the fourth wave of the COVID-19 pandemic. For the present study, answers of a subsample of 848 POC (T1:536, T2: 312) (variables: profession: physician; workplace: outpatient practice or ambulatory medical supply center), assessed via a self-report online questionnaire during the first two waves of the COVID-19 pandemic, were analysed. To minimize response bias at T2, participants that have already participated at T1 were excluded from this survey (*N* = 40). The samples of POC at T1 and T2 were compared with a control sample of 458 PIC (T1: *n* = 262, T2: *n* = 196). The latter was randomly drawn from the total subsamples of PIC (*N* = 1055 at T1, *N* = 959 at T2, total N of PIC = 2014), in order to ensure a similar age and gender structure of the two comparative groups (POC/PIC at T1, POC/PIC at T2) at each time point.

The sample of the total prospective study “VOICE” consisted of 8088 participating HCW during the first time point (T1), between April 20th and July 5th 2020, and 7202 HCW at T2, between November 17th 2020 and January 7th 2021. The total group comprised HCW from diverse professional backgrounds, among them physicians, nurses, medical technical assistants/ MTAs, psychologists and administrative staff. Participants were mainly recruited among the medical staff of the university hospitals of Erlangen, Bonn, Ulm, Cologne, and Dresden. For this purpose, a link for the online survey was distributed via online platform or mailing list (except clinic Ulm). The anonymous 15-min survey including 77 items in German language could be accessed via two academic online survey tools, Unipark (www.unipark.com) and SoSci Survey (www.soscisurvey.de). General inclusion criteria were a minimum age of 18 years, working in the health care sector, residence/ working place in Germany, and sufficient German language skills. The study was approved by the Ethics Committee of the Medical Faculty of the Rheinische Friedrich Wilhem University Bonn (reference number: 125_20) and Medical Faculty of the Friedrich-Alexander University Erlangen-Nürnberg (FAU) (reference number: 133_20 B) and registered on ClinicalTrials (DRKS-ID: DRKS00021268). The date of first registration was 20/04/ 2020. All respondents provided their online informed consent.

#### Outcome measures of mental distress and perceived quality of life

For the present study, primary outcomes were self-assessed current burden, symptoms of depression, anxiety and the subjectively perceived quality of life, at two time points during the COVID-19 pandemic.

*Symptoms of depression and anxiety* were measured using the PHQ-4 (Patient Health Questionnaire) [40]. This ultrashort form (4 items) of the Patient Health Questionnaire (PHQ_D) was divided into two separate modules (PHQ-2 and GAD-2). The PHQ-2 measures depression levels (e.g. „How often did you feel down, depressed, or hopeless over the last two weeks? “), whereas the GAD-2 measures generalized anxiety (e.g. „How often did you feel nervous, anxious or on edge over the last two weeks? “), both with two items and answers ranging from 0 („not at all “) to 3 („nearly every day “). The aggregated sum score for each module ranged from 0 to 6. A cut-off value from ≥ 3 for each module has been suggested to identify likely cases of depression or anxiety. The psychometric characteristics of the PHQ-4 are well documented [40]. In the present sample, the validated German version obtained acceptable Cronbach's Alpha scores of 0.805 for the PHQ-2 and 0.803 for the GAD-2.

*The current burden* level was assessed on a single-item basis. Participants were asked “How much burden did you feel due to the COVID-19 pandemic in the last two weeks?”. An additional item assessed burden retrospectively, before the COVID-19 pandemic (“How much burden did you feel before the COVID-19 pandemic?”). The Likert-type scale ranged from 0 ("not at all") to 4 ("very strong").

*Quality of life (QoL)* was measured with a single item („How would you rate your perceived overall quality of life? “), with answers ranging from 1 (“very bad”) to 5 (“very good”). The complete questionnaire World Health Organizsation Quality of Life (WHOQOL)-BREF is a self-assessment instrument consisting of four domains (physical health, mental health, social relations and environment) and allows a global self-assessment. In our study, we included one question on perceived quality of life: “How would you rate your quality of life today?” Answers could be given on a 5-point Likert scale (1 = very bad to 5 = very good) [41].

#### Sociodemographic, work- and COVID-19-related variables

The online questionnaire assessed general sociodemographic variables, out of which we included age, gender, having children and caring for relatives. In addition, work-related variables such as work-experience, working full-time/part-time, working in home office and change of department were considered. As COVID-19- related variables, the following control variables were of interest in the present study: contact with COVID-19 (having direct contact with infected patients and/or contaminated material), belonging to an at-risk group for a severe infection (due to age or preexisting illness) and previous infection with the COVID-19 virus.

#### Score-Variables assessed as potential burden or resource of POC and PIC throughout the pandemic

*Work family conflict (WFC)/ Family work conflict (FWC)* (according to [42]) were asssessed using four items (e.g. „My work causes burden that makes it difficult to fulfil my family obligations.”) which were rated on a 5-point likert scale (range from 1: “not at all” to 5: “yes, absolutely right”). The overall sum score (min 4, max 20) was used.

*General optimism* (according to [43]) during the COVID-19 pandemic was measured using a single item (“How optimistic have you felt due to the COVID-19 pandemic over the last two weeks?”) with answers ranging from 1 to 7.

*Social Support (ESSI-D)* was measured using the German version of the ENRICHD Social Support Inventory (ESSI-D) [44]. The ESSI is a five-item questionnaire with a score ranging from 5 to 25. A cut-off value of ≤ 18 and the answer of at least two items ≤ 3 are indicative of low social support [45] (The Enriched Investigators 2000). Kendel et al. reported a Cronbach’s alpha of 0.89 for the ESSI, which is in line with the Cronbach’s alpha score of the present sample (0.899).

#### Consent-Variables assessed as potential burden or resource of POC and PIC throughout the pandemic

*Potential COVID-19-related risks and resources during the COVID-19 pandemic* were measured using 15 items (16 at T2) on a scale from 0 ("strongly disagree") to 4 ("strongly agree") with regard to the last two weeks at both time points [46]. At T2, an additional Item 16 „I felt impaired by the restriction of social contact/spare time options.“ was assessed.

*Potential work-related risks and resources during the COVID-19 pandemic* were assessed with six items at T1 and seven items at T2. The items were rated on a scale ranging from 0 "strongly disagree" to 4 "strongly agree" and referred to the past two weeks (e.g. “There is sufficient personnel protective equipment for the staff (including mouth protection)”). At T2, item 3 („I work less than before the pandemic.“) was replaced by („I feel sufficiently informed about the pandemic “) and Item 7 („Today I feel more informed about the pandemic than in spring.“) was added. Consent „yes “ was suggested, if participants had quoted either 3 („rather agree “) or 4 (“strongly agree “).

### Statistical analyses

All statistical analyses were conducted with SPSS Version 28*.* Descriptive statistics (relative frequencies for categorical variables) were calculated to describe the sociodemographic characteristics of the study population (POC) and the control group (PIC). Cross-sectional comparisons with the control group and between the different cohorts of POC at T1 and T2 were performed with the two sample *t*-test for continuous variables or χ^2^-test for categorial variables (Consent: yes/no). The effect sizes (Cohen's d/ Cd and Cramer´s V, CV) were also reported (d ≥ 0.2 = small, d ≥ 0.5 = medium and d ≥ 0.8 = large effect size; V ≥ 0.1 = small, V ≥ 0.3 = medium and V ≥ 0.5 = large effect size) [47]. In order to face the problem of multiple testing, we used Bonferroni-corrected *p*-values (p/number of tests).The prerequisite of normal distribution was tested using Kolmogorov–Smirnov tests. In case of violation against normality or in case of ordinal data, non-parametric testing was preferred (e.g. Spearman´s rank correlations).

A hierarchical three-step, hierarchical multiple linear regression model was calculated for each dependent variable (current burden, PHQ-2, GAD-2, Quality of life) for the sample of POC at T2. To account for confounding factors, significantly correlating sociodemographic, occupational or COVID-19-related control variables were considered in the regression analysis. The sociodemographic variables age, gender, having children and caring for relatives were of interest. In addition, work-related variables such as work-experience, working full-time/part-time, working in home office and change of department were considered. In terms of COVID-19 related variables, the following control variables were of interest in the present study: having direct contact with COVID-19 infected patients, and/or contaminated material, belonging to an at-risk group for a severe SARS-CoV-2-infection (due to age or preexisting illness) and previous infection with the SARS -CoV-2. Variables with significant correlation with the respective outcome variable (see Table S1 of supplementary materials) were entered first (Step 1). Secondly, variables of interest in terms of emotional human relations (WFC, fear of infecting relatives, fear of triage situations, fear patients could die without contact to relatives, worrying about security of patients adversely affected, burden due to restricted social contact in spare time) were entered simultaneously into the regression model. In the third step, variables of interest in terms of supportive human relations (ESSI-D, trust in colleagues, feeling protected through local authorities, feeling protected through employer) were entered simultaneously into the regression model. The influence of each variable was assessed by using the standardized β-coefficient and *p*-value. Since the above mentioned sociodemographic, work- and COVID-19-related control variables were not the focus of the analysis, only the coefficients and test statistics from the third step (except for the adiusted R^2^ of each single step) are presented in the tables. A negative β-coefficient should indicate a protective effect of the respective resource on mental health. Especially in large data sets, the performance of linear regression has been shown to be robust [48]. A level of significance of *p* < 0.05 (two-tailed) was determined in all analyses. Diagnosis concerning multicollinearity of variables was taken into consideration using variance inflation factors and tolerance as indicators. Multicollinearity was not existent in any regression analysis (T2: tolerance: ≥ 0.712, VIF ≤ 1.404).

## Results

### Description of the study sample

A total of 848 physicians working in the outpatient care (POC), assessed during two waves (T1: *n* = 536, T2: *n* = 312), were compared to a sample of 458 physicians working in the inpatient care (PIC) (T1: 262, T2: 196). In sum, in terms of sociodemographic, work- and covid-related control-variables at T1, POC and PIC were comparable (Bonferoni-corrected, see Table 1). At T2, more POC had work-experience of more than six years (96.5% vs. 88.8%) (Cramer´s V = .182, *p* < .001) and more POC had contact with COVID-19 (patients or contaminated material) (Cramer´s V = .174, *p* < .001). In terms of all other control variables (gender, age-group, care for relatives, having children, professional experience, full-time/part-time, home office, change of department, being at risk for a severe COVID-19 infection or having had a COVID-19 infection), POC and PIC were also comparable at T2. For further measures of sociodemographic, work- and COVID-19 related specifications and comparability of the samples PIC/POC at T1 and T2 see Table 1. For measures of comparability of PIC at T1 and T2 and POC at T1 and T2 see Table S2 of the Supplementary material.

**Table 1:** Description and comparability of the study subsamples

Time point	T1			T2		
**Variables of interest/ Control-variables**	**POC** n(%) = 536(100)	**PIC** n(%) = 262 (100)	**POC/PIC** p Cramer`s V	**POC** n (%) = 312 (100)	**PIC (T2)** n (%) = 196	**POC/PIC T2** p Cramer`s V
**Gender**						
Male	216 (40.3)	129 (49.2)	.017	126 (40.4)	101 (51.5)	.038
Female	320 (59.7)	133 (50.8)	.085	185 (59.3)	95 (48.5)	.113
Diverse	0 (0%)			1 (0.3)		
**Age-group in years**						
18–30	6 (1.1)	3 (1.1)	.998	1 (0.3)	1 (0.5)	.513
31–40	51 (9.5)	25 (9.5)	.018	19 (6.1)	12 (6.1)	.092
41–50	137 (25.6)	67 (25.6)		57 (18.3)	37 (18.9)	
51–60	219 (40.9)	108 (41.2)		151 (48.4)	98 (50.0)	
61–70	112 (20.9)	55 (21.0)		71 (22.8)	46 (23.5)	
> 70	11 (2.1)	5 (1.5)		13 (4.2)	2 (1.0)	
**Care for relatives**						
Yes, own household	23 (4.3)	8 (3.1)	.276	15 (4.8)	6 (3.1)	.282
Yes, not own household	86 (16.0)	33 (12.6)	.057	69 (22.1)	35 (17.9)	.071
No	427 (79.7)	221 (84.4)		228 (73.1)	155 (79.1)	
**Children**						
Yes own household	261 (48.7)	127 (48.5)	.397	133 (42.6)	98 (50.0)	.035
Yes not own household	168 (31.3)	73 (27.9)	.048	126 (40.4)	57 (29.1)	.115
No	107 (20.0)	62 (23.7)		53 (17.0)	41 (20.9)	
**Professional experience**						
< 3 years	14 (2.6)	6 (2.3)	.029	3 (1.0)	4 (2.0)	< .001*
3–6 years	14 (2.6)	9 (3.4)	.106	7 (2.2)	7 (3.6)	.182
> 6 years	499 (93.1)	233 (88.9)		301 (96.5)	174 (88.8)	
Not direct patient care	9 (1.7)	14 (5.3)		1 (0.3)	11 (5.6)	
**Full-time/Part-time**						
Full-time	428 (79.9)	191 (72.9)	.027	255 (81.7)	141 (71.9)	.010
Part-time	108 (20.1)	71 (27.1)	.078	57 (18.3)	55 (28.1)	.115
**Homeoffice**						
Yes completely	18 (3.4)	2 (0.8)	.021	1 (0.3)	0 (0.0)	.595
In parts	119 (22.2)	46 (17.6)	.099	51 (16.3)	28 (14.3)	.045
No	399 (74.4)	214 (81.7)		260 (83.3)	168 (85.7)	
**Change of department**						
Yes	66 (12.4)	43 (16.5)	.223	17 (5.5)	21 (10.8)	.027
No	466 (87.4)	217 (83.5)	.062	293 (94.5)	173 (89.2)	.098
**Contact with COVID-19** (infected patients and/or contaminated material)						
Yes	259 (48.6)	151 (58.1)	.012	237 (76.5)	116 (60.1)	< .001*
No	274 (51.4)	109 (41.9)	.089	73 (23.5)	77 (39.9)	.174
**Being at risk**						
Yes	225 (42.3)	103 (39.6)	.472	156 (50.3)	92 (47.4)	.526
No	307 (57.7)	157 (60.4)	.026	154 (49.7)	102 (52.6)	.028
**Infection**						
Yes	4 (.9)	6 (2.3)	.102	8 (2.6)	8 (4.1)	.618
No	345 (64.8)	152 (58.5)	.088	245 (79.0)	151 (78.2)	.044
Don´t know	182 (34.2)	102 (39.2)		57 (18.4)	34 (17.6)	

^*^significance after Bonferroni-correction (p ≤ .05/11 = .005)

### Perceived burden

At T1, POC showed higher scores of Current Burden (Cohen´s d/ Cd = 1.035, *p* = .034), PHQ-2 (Cd = 1.394, *p* = .027) and GAD-2 (Cd = 1.526, *p* = .028), which however were not significant after Bonferoni-correction. POC less often agreed with a higher workload (Cramer´s V/ CV = .099, *p* = .008) (not significant after Bonferoni-correction) and more often reported a reduced workload (CV = .126, *p* < .001). They felt more often burdened through change of tasks (CV = .169, *p* < .001). For further details see Table 2 for variables with medians of sumscores and Table 3 for variables assessed by percentages of consent.

**Table 2:** Perceived burden of POC and PIC T1/T2 and comparison POC T1/T2

**Variable of interest**	**POC** Mean T1 *N* = 536	**POC/PIC T1**	**PIC** Mean T1 *N* = 262	**P (T-Test)**	**Cohen´s d**	**POC** Mean T2 *N* = 312	**POC/PIC T2**	**PIC** Mean T2 *N* = 196	**P (T-Test)**	**Cohen`s d**	**POCT1/T2**	**P (T-Test)**	**Cohen´s d**
Current Burden	**1.42**		1.26	.034	1.035	**1.85**	** > **	1.45	< .001*	.934	** < **	< .001*	.976
Burden before Covid	**.61**		.63	.726	.888	-	-	-	-	-	-	-	-
PHQ-2	**1.58**		1.34	.027	1.394	**2.30**	** > **	1.57	< .001*	1.648	** < **	< .001*	1.580
> Cut off clinical Depression %	**19.0**		16.5	.393	.031 (CV)	**36.1**	** > **	21.3	< .001*	.163 (CV)	<	< .001*	.187 (CV)
GAD-2	**1.68**		1.43	.028	1.526	**2.28**	** > **	1.52	< 001*	1.745	** < **	< .001*	1.700
> Cut off clinical Anxiety in %	**25.5**		20.1	.101	.060 (CV)	**36.8**	>	24.0	< .001*	.139 (CV)	<	< .001*	.120 (CV)
Work family conflict (WFC)	**10.25**		10.42	.630	4.428	**12.23**	** > **	10.88	< .001*	4.170	** < **	< .001*	4.311

^*^ = significance after Bonferoni-Correction: (*p* <  = .05/15) = .003, cut off (PHQ-2, GAD-2) for clinically relevant depression/anxiety >  = 3, CV = Cramer`s V, GAD-2 = separate module of the PHQ-4 (=(Patient Health Questionnaire) assessing anxiety, PHQ-2 = separate module of the PHQ-4 (= Patient Health Questionnaire) assessing depression, *POC* Physicians in the outpatient care, *PIC* Physicians in the inpatient care, *QoL* Quality of Life, *T1* timepoint one of survey, *T2* timepoint two of survey, “- “: not analyzed at T2, “ > “ = significant decrease, “ < “ = significant increase

**Table 3:** Consent with burden in terms of Covid- and work-related problems

**Variable of interest**	**POC T1** Consent %	**POC/PICT1**	**PIC T1** Consent %	**p**	**Cramer`s V**	**POC T2** Consent %	**POC/PICT2**	**PIC T2** Consent %	**p**	**Cramer`s V**	**POCT1/T2**	**p**	**Cramer `s V**
**work-related**													
Increased workload	**21.3**		30.7	.008	.099	**62.4**	** > **	54.1	.108	.078	** < **	< .001*	.411
Reduced workload	**47.5**	** > **	33.7	< .001*	.126	**-**	-	-	-	-	-	-	-
**COVID-19 related**													
Fear of infection	**26.5**		27.0	.893	.005	**40.5**		35.3	.253	.052	** < **	< .001*	.145
Fear infecting others	**40.0**		44.5	.233	.043	**55.3**		50.8	.336	.043	** < **	.001*	.148
Burden due to increased workload	**21.1**		24.6	.267	.040	**58.6**	** > **	41.2	< .001*	.169	** < **	< .001*	.379
Burden due to change of tasks	**51.8**	** > **	34.0	< .001*	.169	**59.5**	** > **	24.1	< .001*	.346		.032	.075
Timidity to work	**9.1**		8.2	.682	.015	**12.5**		8.0	.120	.070		.122	.054
Insomnia	**27.1**		25.4	.617	.018	**41.8**	** > **	29.4	.006	.124	** < **	< .001*	.151
Physical & mental exhaustion	**39.5**		36.7	.461	.026	**66.4**	** > **	38.5	< .001*	.273	** < **	< .001*	.261
Fear of triage situations	**8.7**		8.2	.815	.008	**11.2**		11.2	.988	.001		.245	.041
Fear patients could die without relatives	**30.6**	** > **	25.8	.168	.050	**39.8**		32.1	.085	.078		.007	.094
Security of patients affected	**13.7**		16.8	.258	.041	**34.9**		32.6	.610	.023	** < **	< .001*	.248
Increase of smoking	**6.0**		4.3	.327	.035	**4.6**		5.9	.532	.028		.398	.030
Increase of alcohol intake	**13.3**		13.3	.980	.001	**16.1**		12.8	.321	.045		.274	.038
Increase of intake of antidepressants	**1.9**		3.1	.302	.037	**7.2**		1.6	.006	.124	** < **	< .001*	.132
Burden due to restricted social contacts	**-**		-	-	-	**72.2**		71.4	.298	.051		-	-

*POC* Physicians in the outpatient care, *PIC* Physicians in the inpatient care, *T1* timepoint one of survey, *T2* timepoint two of survey Consent %: (rate in % of: sum of 3: rather agree + 4: fully agree on a 5 point Likert-Scale (0-4)), “- “ = not assessed at T1/T2 “ > “ = significant decrease, “ < “ = significant increase, *significance after Bonferoni-Correction: (.05/27) = .002

At T2, POC showed significantly higher levels of nearly any score parameter of burden, assessed at a level of significance of *p* < .001, compared with PIC. In addition, POC also agreed more often with a burden due to increased workload (CV = .169, *p* < .001), due to change of tasks (CV = .346, *p* < .001), insomnia (CV = .124, *p* = .006) (not significant after Bonferoni-correction), physical and mental exhaustion (CV = .273, *p* < .001) and felt less informed (CV = .209, *p* < .001). For further details see Table 2 for variables with medians of sumscores and Table 3 for variables assessed by percentages of consent.

**Fig. 1:**
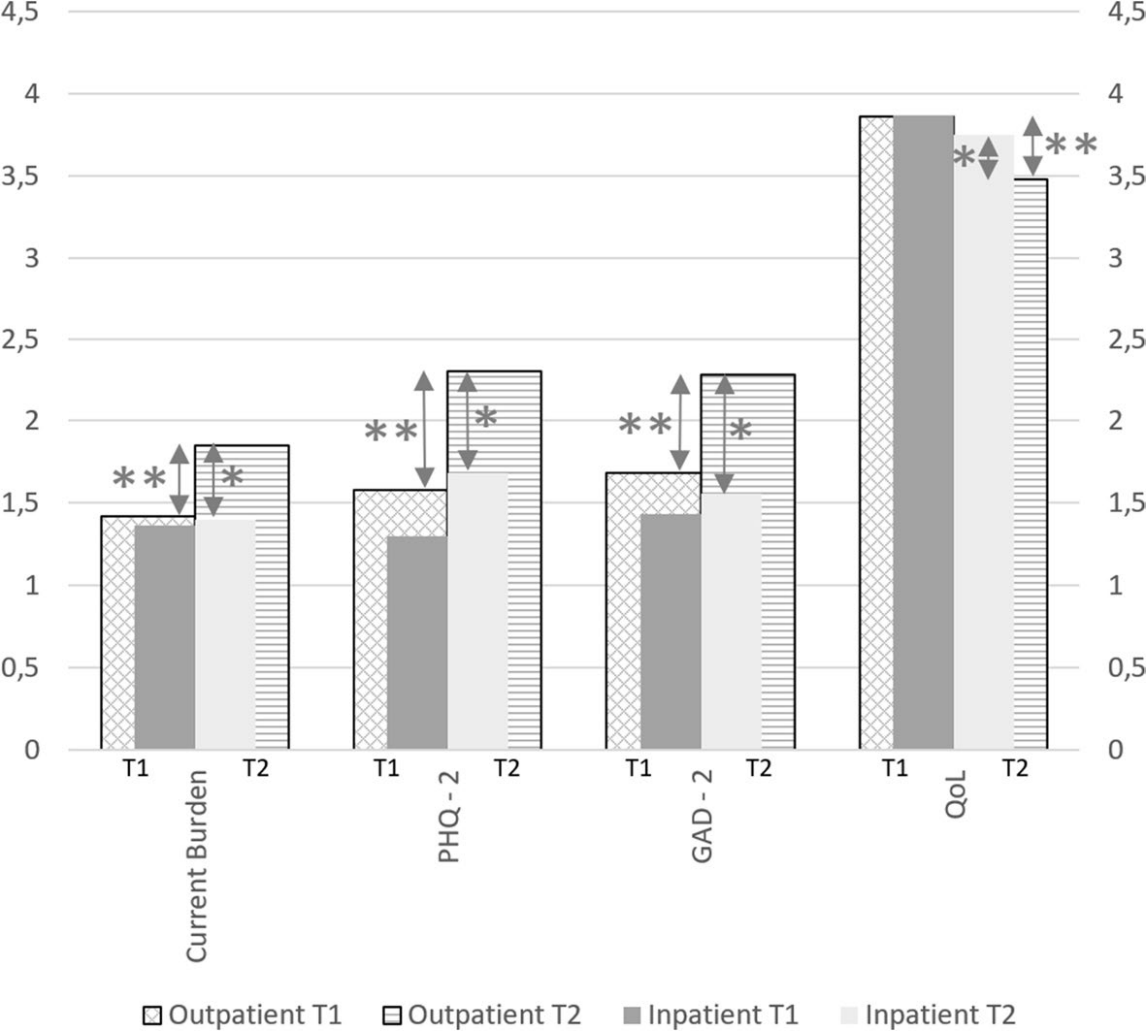
Visualization of Main Outcomes (Current Burden, PHQ-2, GAD-2 and QoL) of POC and PIC at T1 and T2**.** PHQ-2 = separate module of the PHQ-4 = (Patient Health Questionnaire) assessing depression, GAD-2 = separate module of the PHQ-4 = (Patient Health Questionnaire) assessing anxiety, QoL = Quality of life, POC = Physicians in outpatient care, PIC = Physicians in inpatient care, T1 = timepoint one of the survey, T2 = timepoint two of the survey, * significant difference POC/PIC T2 (*p* < .001), ** significant difference POCT1/POCT2 (*p* < .001)

Throughout the pandemic (T2 compared with T1), all parameters of burden assessed increased at a level of significance (*p* < .001) within their own cohort of POC. At T2, POC reported an increased workload (CV = .411, *p* < .001), fear of infection (CV = .145, *p* < .001), fear of infecting others (CV = .148, *p* = .001), burden due to increased workload (CV = .379, *p* < .001), insomnia (CV = .151, *p* < .001), physical and mental exhaustion (CV = .261, *p* < .001), worrying about the patient`s security (CV = .248, *p* < .001) and intake of antidepressants (CV = .132, *p* < .001). For further details see Table 2 for variables with medians of sumscores and Table 3 for variables assessed by percentages of consent. For a visualization of the comparison between POC/PIC at T1 and T2, and the course of the outcome parameters/parameters of burden throughout the pandemic see Fig. 1.

### Resources

At T1, POC significantly less often agreed with having sufficient protective gear (CV = .152, *p* < .001), but more often with having sufficient staff (CV = .123, *p* < .001). POC felt less often protected by local authorities (CV = .156, *p* < .001). At T2, they reported lower ESSI-D (Cd = 4.318, *p* = .002), Quality of life (Cd = .891, *p* = .002). At T2, they less often felt sufficiently informed (CV = .209, *p* < .001), and felt less often protected by local authorities (CV = .183, *p* < .001) than PIC. Throughout the pandemic within their cohort, quality of life was reduced (Cd = .865, *p* < .001) as well as ESSI-D (Cd = 4.195, *p* < .001), while consent with being supplied by protective gear (CV = 0.366, *p* < .001) were increased. Consent with having sufficient staff decreased (CV = .359, *p* < .001) as well as sufficient recreation during spare time (CV = .217, *p* < .001) and with feeling protected by local authorities (CV = .132, *p* < .001). For further details see Table 4 for variables with medians of sumscores and Table 5 for variables assessed by percentages of consent.

**Table 4:** Comparison of Resources between POC and PIC at T1 and T2

**Variables of interest**	**POC** Mean T1	**POC/PICT1**	**PIC** Mean T1 **PIC** Mean T1 **PIC** Mean T1	**P (T-Test)**	**Cohen`s d**	**POC** Mean T2	**POC/PICT2**	**PIC** Mean T2	**P (T-Test)**	**Cohen`s d**	**POC T1/T2**	**P (T-Test)**	**Cohen`s d**
General Optimism	**5.23**		5.05	.096	1.368	**5.09**		5.31	.090	1.374		.199	1.391
QoL	**3.86**		3.87	.961	.827	**3.48**	<	3.74	.002	.891	>	< .001*	.865
ESSI-D	**20.49**		20.55	0.836	3.860	**19.32**		20.58	.002*	4,318	>	< .001*	4.195

^*^ = significance after Bonferoni-Correction: (*p* <  = .05/17) = .003, CV = Cramer`s V, *QoL* Quality of life, “ > “ = significant decrease, “ < “ = significant increase

**Table 5:** Consent with resources in terms of covid- and work-related variables

**Variable of interest** Consent with:	**POCT1** Consent %	**POC/PICT1**	**PICT1** Consent %	**p**	**TCramer`s V**	**POCT2** Consent %	**POC:PIC T2**	**PICT2** Consent %	**p**	**Cramer`s V**	**POC T1/T2**	**p**	**Cramer`s V**
**Work related variables**													
Protective gear	**31.6**	** < **	47.8	< .001*	.152	**69.5**		81.2	.011	.122	** < **	< .001*	.366
Sufficient Information	**-**	-	-	-	-	**66.2**	** < **	85.3	< .001*	.209	-	-	-
Sufficient staff	**73.4**	** > **	61.0	< .001*	.123	**36.9**		30.1	.167	.067	** > **	< .001*	.359
Sufficient recovery during spare time	**41.7**		40.5	.772	.011	**20.3**	** < **	27.8	.087	.083	** > **	< .001*	.217
Trust in colleagues	**68.8**		70.0	.767	.011	**67.1**		76.7	.045	.097		.621	.018
Better degree of information than in spring	**-**		-	-	-	**52.2**		57.1	.343	.046		-	-
**Covid-related variables**													
Protection by local authorities	**28.4**	** < **	44.1	< .001*	.156	**16.8**	** < **	32.6	< .001*	.183	** > **	< .001*	.132
Protection by employer	**47.0**		46.5	.892	.005	**47.0**		51.9	.298	.047		.992	.000

*POC* Physicians in the outpatient care, *PIC* Physicians in the inpatient care, *T1* timepoint one of survey, *T2* timepoint two of survey, Consent %: (rate in % of: sum of 3: rather agree +45: fully agree on a 5 point Likert-Scale (0-4)), *QoL* Quality of life, * = significance after Bonferoni-Correction: (.05/27) = .002, “- “ = not assessed at T1/T2, “ > “ = significant decrease, “ < “ = significant increase

### Regression of current burden, depression (PHQ-2), anxiety (GAD-2) and perceived Quality of life (QoL) on emotional and supportive human relations at T2

#### Current burden

Risk factors for increased burden at T2 were work-family conflict (WFC) (ß = .254, *p* < .001, 95% CI: .23, .28), worrying about patients security (ß = .144, *p* = .007, 95% CI: .07, .22) and burden through restricted social contacts (ß = .146, *p* = .003, 95% CI: .07, .22). Protective factor was feeling protected by local authorities (ß = -.302, *p* < .001, 95% CI: -.39, -.22). The present model explained 45.4% of the variance. Total of entered variables of supportive human relations added 11.5% to a variance explained by sociodemographics and variables of emotional human relations (33.9%). None of the entered control variables (working full-time/part-time, contact with COVID-19) were significant. For further details see Table 6.

**Table 6:** Hierarchical regression analysis of control variables, variables of emotional and supportive human relations and outcome variables current burden and severity of depressive symptoms (PHQ-2) for the sample of POC at T2 (*n* = 312)

Outcome variable Variable of interest	Current Burden					PHQ-2				
	B (95% CI)	SE	β (95% CI)	T	P	B (95% CI)	SE	β (95% CI)	T	p
**Constant**	2.873 (2.14, 3.61)	.373		7.710	< .001*	4.252 (2.62, 5.88)	.827		5.143	< .001*
**Step 1 Control variable** (with a significant correlation *p* < = .05)	Adj. *R*^*2*^ = .080					Adj. *R*^*2*^ = .028				
Having Children	-	-	-	-	-	.220 (-.02, .46)	.120	.090 (-.15, .33)	1.827	.069
Working fulltime/parttime	-.174 (-.40, .05)	.115	-.071 (-.30, .15)	-1.512	.132	.112 (-.34, .56)	.228	.024 (-.42, .47)	.492	.623
Contact-COVID	.134 (-.08, .35)	.110	.059 (-.16, .27)	1.219	.224	-	-	-	-	-
**Step 2 Emotional human relations**	Adj. *R*^*2*^ = .339					Adj. *R*^*2*^ = ..283				
WFC	.058 (.04, .08)	.012	.254 (.23, .28)	4.902	< .001*	.061 (.01, .11)	.024	.139 (.09, .19)	2.569	.011*
Fear contagion of relatives	.038 (-.03, .11)	.036	.051 (-.02, .12)	1.059	.291	.006 (-.13, .14)	.070	.004 (-.13, .14)	.082	.935
Triage	.043 (-.04, .13)	.042	.049 (-.03, .13)	1.036	.301	.134 (-.03, .30)	.082	.081 (-.08, .24)	1.625	.105
Fear patients die without contact relatives	.045 (-.02, .11)	.032	.068 (.01, .13)	1.399	.163	.087 (-.04, .21)	.064	.068 (-.06, .19)	1.364	.174
Pat. security adversely affected	.104 (.03, .18)	.038	.144 (.07, .22)	2.704	.007*	.206 (.06, .35)	.075	.150 (.00, .30)	2.748	.006*
Burden due to restricted social contacts	.119 (.04, .20)	.040	.146 (.07, .22)	2.989	.003*	.290 (.14, .44)	.078	.187 (.03, .34)	3.700	< .001*
**Step 3 supportive human relations**	Adj. *R*^*2*^ = .454					Adj. *R*^*2*^ = ..413				
ESSI-D	.009 (-.01, .03)	.010	.044 (.02, .06)	.918	.359	-.072 (-.11, -.03)	.020	-.180 (-.22, -.14)	-3.594	< .001*
Trust in colleagues	-.068 (-.15, .01)	.042	-.079 (-.16, .00)	-1.632	.104	-.294 (-.46, -.13)	.082	-.181 (-.34, -.02)	-3.599	< .001*
Protection by local authorities	-.266 (-.35, -.18)	.044	-.302 (-.39, -.22)	-5.999	< .001*	-.317 (-.49, -.15)	.087	-.190 (-.36, -.02)	-3.640	< .001*
Protection by employer	-.005 (-.07, .06)	.033	-.007 (-.07, .06)	-.158	.875	-.039 (-.17, .09)	.064	-.029 (-.15, .10)	-.605	.546
*Adj. R*^*2*^ adjusted R^2^ (explained variance), *ESSI-D* ENRICHD Social Support Inventory, *SE* standard error, *WFC* Work Family Conflict, “- “ = not entered into regression analyses due to no significant correlation between control variable and considered outcome parameter (p > .05, see description of statistical analyses above), significant *p*-values are marked (*)										

#### PHQ-2

Risk factors for depression (PHQ-2) at T2 were WFC (ß = .139, *p* = .011, 95% CI: .09, .19), worrying about patients’ security (ß = .150, *p* = .006, 95% CI: .00, .30) and burden due to restricted social contacts during spare time (ß = .187, *p* < .001, 95% CI: .03, .34). Protective factors were Social Support (ESSI-D) (ß = -.180, *p* < .001, 95% CI: -.22, -.14), trust in colleagues (ß = -.180, *p* < .001, 95% CI: -.34, -.02) and feeling protected by local authorities (ß = -.190, *p* < .001, 95% CI: -.36, -.02). The present model explained 41.3% of the variance. Total of entered variables of supportive human relations added 13.0% to a variance explained by sociodemographics and variables of emotional human relations (28.3%). None of the entered control variables (having children, working full-time/part-time) were significant. For further details see Table 6.

#### GAD-2

Risk factors for anxiety (GAD-2) at T2 were WFC (ß = .207, *p* < .001, 95% CI: .16, .26), fear to decide who gets care and who not (triage situations) (ß = .132, *p* = .010, 95% CI: -.04, .31) and burden due to restricted social contacts during spare time (ß = .156, *p* = .003, 95% CI: -.01, .32). Protective factors were Social Support (ESSI-D) (ß = -.127, *p* = .014, CI 95%: -.17, -.08), trust in colleagues (ß = -.199, *p* < .001, 95% CI: -.37, 0.02) and feeling protected by local authorities (ß = -.211, *p* < .001, 95% CI: -.40, -.03). None of the entered control variables (working full-time/part-time, contact with infected, contact with contaminated material) were significant. The present model explained 37.8% of the variance. Total of entered variables of supportive human relations added 11.9% to a variance explained by sociodemographic/ work-related variables and variables of emotional human relations (25.9%). None of the entered control variables (working full-time/part-time, contact with COVID-19) were significant. For further details see Table 7.

**Table 7:** Hierarchical regression analysis of control variables, variables of emotional and supportive human relations and outcome variables GAD-2 and QoL at T2 for the sample of POC at T2 (*n* = 312)

Outcome variable Variable of interest	**GAD-2**					**QoL**				
	B (95% CI)	SE	β (95% CI)	T	p	B (95% CI)	SE	β (95% CI)	T	p
Constant	4.015 (2.45, 5.58)	.794		5.056	< .001*	2.981 (2.21, 3.75)	.392		7.605	< .001*
**Step 1 Control variables** (with a significant corellation p < = .05)	Adj. *R*^*2*^ = .029					Adj. *R*^*2*^ = .012				
Having children	-	-	-	-	-	-.105 (-.23, .02)	.062	-.083 (-.20, .04)	-1.686	.093
Fulltime/Parttime	.132 (-.35, .62)	.246	.027 (-.46, .51)	.536	.593	-	-	-	-	-
Contact COVID	.399 (-.06, .86)	.234	.088 (-.37, .55)	1.704	.090	-	-	-	-	-
**Step 2 Emotional human relations**	Adj. *R*^*2*^ = .259					Adj. *R*^*2*^ = .223				
WFC	.095 (.05, .15)	.025	.207 (.16, .26)	3.733	< .001*	-.046 (-.07, -.02)	.012	-.207 (-.24, -.18)	-3.766	< .001*
Fear of infecting relatives	.032 (-.12, .18)	.076	.022 (-.13, .17)	.425	.671	-.018 (-.09, .06)	.037	-.025 (-.10, .05)	-.480	.632
Fear of triage situations	.232 (.06, .41)	.089	.132 (-.04, .31)	2.599	.010*	-.061 (-.15, .03)	.043	-.071 (-.16, .01)	-1.404	.162
Fear patients die w/out contact to relatives	-.007 (-.14, .13)	.069	-.005 (-.14, .13)	-.100	.920	.008 (-.06, .07)	.033	.012 (-.05, .08)	.234	.815
Fear patients security adversely affected	.112 (-.05, .27)	.082	.078 (-.08, .24)	1.362	.174	-.031 (-.11, .05)	.039	-.045 (-.12, .03)	-.794	.428
Burden due to restricted social contacts	.254 (.09, .42)	.085	.156 (-.01, .32)	2.990	.003*	-.098 (-.18, -.02)	.041	-.123 (-.20, -.04)	-2.391	.018*
**Step 3 Supportive human relations**	Adj. *R*^*2*^ = .378					Adj. *R*^*2*^ = .385				
ESSI-D	-.053 (-.10, -.01)	.022	-.127 (-.17, -.08)	-2.465	.014*	.043 (.02 .06)	.011	.211 (.19, .23)	4.105	< .001*
Trust in colleagues	-.340 (-.52, -.17)	.089	-.199 (-.37, -.02)	-3.930	< .001*	.103 (.02, .19)	.043	.124 (.04, .21)	2.398	.017*
Protection by local authorities	-.371 (-.56, -.19)	.094	-.211 (-.40, -.03)	-3.930	< .001*	.235 (.15, .32)	.046	.273 (.18, .36)	5.140	< .001*
Protection by employer	.015 (-.12, .15)	.070	.011 (-.13, .15)	.214	.831	.022 (-.04, .09)	.034	.033 (-.03, .10)	.655	.513

*Adj. R*^*2*^ adjusted R^2^ (explained variance), *ESSI-D* ENRICHD Social Support Inventory, *QoL* Quality of Life, *SE* standard error, *WFC* Work Family Conflict, “- “ = not entered into regression analyses due to no significant correlation between control variable and considered outcome parameter (p > .05, see description of statistical analyses above), significant *p*-values are marked (*)

#### QoL

Factors influencing the perceived Quality of life positively were ESSI-D (ß = .211, *p* < .001, 95% CI: .19, .23), trust in colleagues (ß = .124, *p* = .017, 95% CI. .04, .21) and feeling protected by local authorities (ß = .273, *p* < .001, 95% CI: .18, .36). Challenges for QoL were WFC (ß = -.207, *p* < .001, 95% CI: -.24, -.18) and burden through reduced social contacts during spare time (ß = -.123, *p* = .018, 95% CI: -.20, -.04). The present model explained 38.5% of the variance. The total of entered variables of supportive human relations added 16.2% to a variance explained by control variables and variables of emotional human relations (22.3%). None of the entered control variables (having children) were significant. For further details see Table 7.

## Discussion

Results of the present study on mental distress of physicians in the outpatient care (POC) throughout the COVID-19 pandemic suggested an increased psychosocial burden and at the same time decreased resources among POC throughout the pandemic. Regression models revealed a substantial impact of aspects of emotional and supportive human relations, such as moral and private concerns, as well as perceived occupational trust and support, on mental health and perceived quality of life.

### Mental distress of POC—compared with PIC and throughout the pandemic

A detected comparable subjective current burden of POC at T1 and their inpatient colleagues (PIC) and also comparable burden before COVID-19 suggested that POC did not generally enter the pandemic with a higher „prestrain “ than their colleagues. Therefore, assumably the assessed increase of mental distress at the beginning and thoughout the pandemic is likely to be caused by the prospective circumstances related to the pandemic. In line, POC displayed higher burden through change of tasks. In terms of mental distress of HCW, results of a study in Nepal suggested symptoms of stress, anxiety, and depression of 28.9%, 35.6% and 17.0% respectively [49]. Results for our subgroup of POC suggest slightly higher rates with 19.0% for depression and lower rates with 25.5% for anxiety, during the first wave of the pandemic. Of note, although only 1.4% of POC had been infected with COVID-19 at the beginning of the pandemic, at the same time nearly every fifth POC showed symptoms of depression and over one fourth of POC showed levels of anxiety considered as probably clinically relevant on the basis of established cut-off values [40]. These results support the already published conclusion that the psychosocial footprint of the COVID-19 pandemic is likely to be broader than its purely medical footprint on HCW [36, 50].

However, in terms of workload, one practical reason for the perception of fewer working hours among POC at the beginning of the pandemic could be the substantial drop of patient numbers during the first lockdown in primary care in Germany [33]. Related economic concerns, including worries about (lower) patient numbers, leading directly to existential fears of the POC who are mostly self-employed entrepreneurs [33]. The latter could contribute to a higher burden and may explain slightly higher scores of mental distress compared with PIC. Additionally, our results of a higher perceived burden through change of work-tasks are in line with previous research highlighting organizational changes having a negative impact on psychological distress [51] and changes of tasks as a source of strain of GPs [19]. Previous results of more than one fifth of GPs reporting a deteriorated work climate early in the pandemic led to the assumption of an impact of specific aspects of burden associated with the pandemic, independent of patient numbers and the associated workload [33]. This assumption is supported by our results of a slightly higher burden despite lower workload of POC at the beginning of the pandemic and less trust in local authorities as well as social support, declining throughout the pandemic.

Of note, just a few months later, at the second point of the assessment, POC showed significantly higher levels of nearly all parameters of burden, including higher workload and persisting burden through change of work tasks compared with PIC. In addition, nearly all burden-related parameters assessed increased within the cohort of POC at T1 and T2, suggesting a significant increase of mental distress throughout the pandemic. POC felt less protected by local authorities, more often agreed with physical complaints such as insomnia, physical and mental exhaustion, and worrying about patients’ security adversely affected. These findings support earlier assumptions of an upcoming higher workload for POC due to the delay and accumulation of postponed mandates of non-COVID-19 care, increasing numbers of COVID-19 cases and transfer of COVID-19 care demands to outpatient practices as well as higher administrative workload [33].

### Risk factors of mental distress throughout the pandemic

Even at the beginning of the pandemic, when workload was perceived as lower, POC reported strain in terms of human relations such as higher scores of moral concerns. Particularly, compared with PIC, more POC worried about patients dying without seeing their relatives again. Throughout the pandemic, worrying about the security of patients remained higher compared with PIC and increased within the cohort of POC. In line, previous results of research demonstrated moral concerns of GPs such as worrying about elderly or mentally ill patients, which are due to lockdown restrictions, such as not being able to see their relatives or to have sufficient social interaction [19]. Furthermore, HCW anticipated concerns of collateral damage to the health of the population due to abandoned or postponed routine care [15–17]. Moral issues can play a key role „on top “ of daily worklife challenges during COVID-19 [35, 52]. In fact, the identified differences of burden through moral concerns, in particular worrying about patients dying without contact with relatives between POC and PIC as well as increasing concerns of an adversely affected security of patients, could be related to a longer and more intense relationship between POC and their patients in the outpatient care [53]. Furthermore, supporting previous research, POC were increasingly worrying about getting infected with COVID-19, suggesting GPs to be convinced that they are at high risk for an infection [19]. Generalized anxiety had been found to be associated with the fear of being infected with the virus before, partly explained by unclear pathways of infection and hygiene measures at the beginning of the pandemic [32]. An association between fear of infection/ infecting relatives and mental distress [36, 50] and an inverse association between safety aspects and risk of burnout in HCW during the COVID-19 pandemic has been outlined in HCW [12]. Of note, research has indicated that GPs are rather concerned about infecting others or burdening colleagues by not contributing their part of work when becoming ill themselves [19]. This finding supports the notion that interpersonal aspects might play a superior role over individual factors in terms of mental distress. In terms of individual sociodemographic aspects, research has suggested relatively low evidence for their prominent influencial role on mental distress of HCW [36, 54]. Specifically, throughout the pandemic, only having children was associated with higher depression in our study. Our findings of an increased work-family-conflict being a risk factor for burden, anxiety and low perceived quality of life throughout the pandemic, support the obvious difficulties parents working in the health care sector have balancing work and private life every day. Furthermore, low work experience played a role as a predictor for depression at the beginning, but not later in the pandemic. In this context, the critical practice in clinics of putting the least experienced physicians on the frontline has been mentioned [55]. However, transferibilty of this circumstance into outpatient context remains questionable. Nethertheless, our findings of a considerably low influence of sociodemographic variables support the notion of more influencial variables on differentiating risk groups for depression and anxiety [36, 54].

In terms of supportive human relations throughout the pandemic, POC felt less often supported by local authorities, displayed less trust in colleagues, less social support and perceived quality of life compared to PIC. Above, perceived quality of life, social support and perceived protection through authorities decreased throughout the pandemic within the cohort of POC. Of note, perceived protection by local authorities, trust in colleagues and social support were identified as protective factors for mental distress and quality of life throughout the pandemic. For HCW, aspects of social support had already been previously suggested as protective against increased mental distress [36, 37]. However, stability of social support only within the first months, not in the whole course of the pandemic, was of interest in this case [37]. Our results add a decrease of social support throughout the pandemic, at least for POC. Reasons could be directly related to the features of the pandemic, such as contact restrictions and lock-downs, as well as indirectly caused by burden caused by the pandemic, such as work-family conflict, lacking trust in authorities, employers or colleagues. It seems obvious that burden due to restricted contacts during spare time is cojoined by a decline of perceived social support. But restrictions might be compensated by ongoing existing social contacts within the work team or family [37]. It can be assumed that social recreational opportunities in spare time are perceived as a sufficient recovery. However, both can be negatively influenced by work-family-conflict, substantiated by the circumstance of work-family-conflict as a risk factor for burden and perceived quality of life in the present study. In line, previous results revealed HCW with additional care-giving responsibilities in their family experience higher levels of stress due to increased workload and change of tasks [56]. Furthermore, perceived burden through restrictions of social contacts during spare time was identified as a stable risk factor for mental distress and perceived quality of life, throughout the pandemic. Unfortunately, as this complex interplay cannot be fully distangeled by the results of our cross-sectional investigation, causal conclusions cannot yet be drawn. Longitudinal prospective research on influencial factors of social support would be advantageous to shedding light on causes of this richly layered construct. Yet, results of our study underline an impact of social support as potential resource for mental health and perceived quality of life of HCW and strengthen previous findings [36, 37, 57, 58].

Results of our study revealed a stability of consent with trust in colleagues within the cohort of POC, although they generally scored lower compared to PIC throughout the pandemic. One explanation might be the ‚lone fighther ‘ status of GPs. However, the stability of about three thirds of POC agreeing to be able to trust in colleagues throughout the pandemic supports observations of a lot of solidarity between the different HCW [19]. In line, previous investigations suggest 15.1% of GPs reporting an even improved work climate during the first months of the pandemic [33]. The assumption that due to the lower workload, the work climate between GPs and other medical specialists developed even better and smoother [19], can only be partly supported by our results from the beginning of the pandemic. However, it does not explain the stability of trust in colleagues despite the outrageous increase of workload later during the pandemic. Current findings highlight an increasing solidarity and team cohesion in times of crisis [59, 60]. In fact, some specialists seemed to have more time to exchange information more profoundly, leading to a facilitation of collaboration [19], once more indicating a stable bond of trust in colleagues throughout outpatient care. Our study results identified trust in colleagues and feeling protected by local authorities as protective factors for mental distress and perceived quality of life. It can be speculated that advantageous and disadvantageous influencial factors might sum up to an overall stabiltiy of trust in colleagues among POC. However, results are in line with the previous results of our study group, having identified team aspects and social support as protective factors for HCW in clinical settings or among larger populations of mixed professions [7, 36, 37]. Although POC are usually not integrated in a large team structure common in clinics, team supervisions and encouragement of a healthy team structure are crucial for the outpatient care. Of note, informal exchange with colleagues e.g. during online training courses, was experienced as valuable earlier in the crisis [10].Work climate in general seemed to play an important role in terms of severity of perceived burden at the beginning of the COVID-19 pandemic [33]. In terms of other work related factors, fortunately significantly more POC agreed with being sufficiently equipped with protective gear at T2 than at T1. These results support earlier results of research, whereas the majority of GPs stated still being able to care for their patients properly [33] but claimed to be equipped with the same protective gear as their inpatient colleagues [23, 33]. Interestingly, already at the beginning of the pandemic, POC felt less supported by local authorities indicating GPs displaying a lower degree of information and preparedness [19]. However, while sufficient protective gear as the most important factor influencing pandemic preparedness of general practitioners in Germany has been claimed [61], POC in our study, although apparently better equipped throughout the pandemic, did not automatically feel better informed or better prepared than at T1. The perceived deficit of information and support of local authorities compared with PIC can therefore not be compensated simply as a result of a better perceived equipment status throughout the pandemic, possibly contributing to mental distress [19]. Therefore, aspects of internal communication seem to have a high potential regarding the impact on work climate compared to individual fears or lack of protective materials [33]. In line, despite feeling better equipped with protective gear later in the pandemic, POC still exhibited higher mental distress in nearly all parameters of burden assessed. Moreover, aspects of supportive human relations such as perceived protection by local authorities and trust in colleagues turned out as prominent risk and protective factors of mental distress and perceived quality of life throughout the pandemic. This is in line with the results of earlier studies revealing perceived non-preparedness [19] and an adverse work climate as associated factors with burden throughout the pandemic [33] and are in line with the statements of the GPs complaining about lacking preparedness of authorities. The results of research of our workgroup on a comparably large cohort of different professions of HCW in clinics suggested a lack of trust in one’s own working team as a risk factor for generalized anxiety and depression [7]. The authors pointed towards the impact of interaction at work through cooperation and information exchange, highlighting that belonging to a team encourages feelings of security and self-esteem. Because of the pandemic situation, the demands on the functioning of the team have increased, which might be a reason why a lack of trust in one’s own working group is accompanied by high psychological stress [7].

Throughout the pandemic, perceived quality of life and protection by local authorities and social support again substantially dropped among the two cohorts of POC. However, stability and comparability of optimism, trust in colleagues between the two groups, and stability throughout the pandemic within the cohorts of POC, makes resignation as influential factor for the higher burden of POC rather unlikely. Consistent with the previous research, social support was associated with less depression and less general anxiety symptoms [36].

Interestingly, levels of perceived social support did not differ from the pre-pandemic results [44] and from other professions of HCW [37], even though restrictions had been imposed on social life during the pandemic, leading to an enormous change of conditions of work- and spare time. One explanation for the rather comparable level of subjective social support before and within the first months of the pandemic, could be the persistence of social support in the working teams, together with the ongoing presence of family support at the beginning of the pandemic, which partly compensated for restrictions in terms of private restrictions outside the core-family [37]. Our results throughout the crisis do not completely support this derivation, since the quality of social interaction in terms of family, and perceived protection by authorities seem to worsen, as well as the overall perceived social support (at least for the physicians in the outpatient setting in our sample). The deterioration of all of these items which were identified as risk factors, explains a large part of the variance predicting depression, anxiety burden and perceived quality of life. Although HCW reported normal levels of perceived social support, they might not have been able to engage in social contact as sufficiently as before because of the social restrictions during the pandemic, or the fear of infecting people when meeting in-person [37]. However, although protective effects of social support might be smaller in times of the pandemic, they could still counteract feelings of loneliness and consequently reduce symptoms of depression [62]. This assumption is in line with our study results.

## Conclusion

The present study aimed to investigate mental distress as well as risk and protective factors of mental distress in POC throughout the COVID-19 pandemic. To sum up, as hypothesized initially and based on the scarce findings, our results on POC at two different time points of the pandemic suggest their significant psychosocial burden and its increase throughout the pandemic. More specifically, although describing a lower subjective workload at the beginning of the pandemic, POC displayed higher scores of depression and a higher perceived burden through change of tasks as well as more worrying that patients could die without contact with their relatives. Of note, a comparable burden before COVID-19 suggested that POC did not generally enter the pandemic with a higher „prestrain “ compared with their colleagues. Also, results revealed the affore-hypothesized lower perceived support e.g. through colleagues, employers, local authorities compared to their colleagues in the inpatient care (PIC) Moreover, a rapid decline of perceived social support and subjective quality of life over a few months was confirmed. However, differently of what was supposed, POC exhibited similar resources in terms of general optimism and social support, compared to their colleagues working in clinics. Finally, the supposed impact of existing intraindividual and interindividual risk and protective factors on mental distress and quality of life throughout the pandemic could be confirmed. The substantial finding of the present work is the predominant role of emotional and supportive human relations in terms of interpersonal protective factors for depression, anxiety and quality of life of POC. Among the interpersonal protective factors are feeling protected by local authorities, trust in colleagues and perceived social support. Of note, trust in colleagues remained stable throughout the pandemic and seemed to be one of the key predictors for protection against depression, anxiety and a worsening quality of life. Consequently, investing more into the various, presumably summative influence of the variable trust in colleagues, as well as into other supportive team aspects and the role and course of a supportive private climate, seems to be of great worth. Investing into the strengthening of personal existing resources, and at the same time, offering a comparative amount of attention and effort to protection and support of physicians in both outpatient and inpatient care, may be beneficial for the maintenance of good mental health and work ability. The dominant impact of perceived protection by local authorities over apparently less influencial perceived protection through employer, the degree of information and current availability of protective material such as face masks seems worth to be investigated more thoroughly. As assumed, private emotional interpersonal aspects of burden, such as work and family conflict, and burden due to restricted opportunities and contacts during spare time, as well as moral concerns contribute to mental distress to an even higher extent than COVID-19-, work-related and sociodemographic factors. As a matter of fact, we interpret the homogeneity of the risk and protective factors for mental distress, as well as supportive inverse results for quality of life as additional confirmation for the so far underestimated impact of emotional and supportive interpersonal issues in the long-term course of psychosocial burden of POC.

### Strengths and limitations

To the best of our knowledge, the present survey preliminarily investigates the mental health of large samples of physicians in the outpatient care at two different time points of the COVID-19 pandemic, including a broad variety of assessed variables of burden and resources. Above, a comparison with colleagues in the inpatient setting was realized. However, our study has some limitations. As the survey design is cross-sectional, causal conclusions cannot be drawn.

A further limitation of the study is, that all data were self-reported therefore objective verification is not possible. However, an anonymous character was mandatory for the protection of identities for ethical purposes. Due to the method of data collection, a possible selection bias of the sample must be considered. Another limitation concerns the voluntary nature of our study, which may be related to a response bias. The design only allowed a self-selected sample within the addressed cohorts. Therefore, it also cannot be ruled out, that either especially burdened or especially resourceful physicians participated. Furthermore, comparability of the two study samples (POC and PIC) is limited, since these two very heterogenous groups were faced with different challenges during the COVID-19 pandemic. Therefore results should be treated with caution and analyses with larger sample sizes allowing further stratification are warranted. A longitudinal analysis with the exclusive attention on physicians in the inpatient setting (their overall total cohort is much larger than the age-and gender matched sample used as a control group here) in terms of their mental distress and their emotional and interrelational resources is planned by members of our work-group.

Additionally, due to economic reasons, we assessed data with short versions (e.g. for depression and anxiety) of the original instruments or single item measures (Current Burden and Quality of Life), possibly reducing criterion validity. We are aware that the perception of the own current burden and quality of life illustrate two rather subjective variables vulnerable for a self-assessment bias typical for single items. However, the PHQ-2 and GAD-2 have been well studied and widely used in the past [40].

Another limitation is that for the present study no sample size calculation was done but a post-hoc power analysis. The reason for this is that the present study is part of the VOICE-project with the primary aim to assess mental health during the COVID-19 pandemic in health care workers in general [7]. However, a post-hoc power analysis using G*Power [63], assuming a multiple linear regression model, a medium effect size, type I error of 5% and including 13 predictors, revealed a quitely high power of 96%.

And, although rather unlikely, through the setting of an online questionnaire, a social desirabilty cannot be completely ruled out. In addition, although the compared groups of interest came from a different work-setting, we did not additionally recruit a control group from the general population. Even though the data suggest that for instance the resources attenuate mental health problems, other interpretations could be possible. Therefore, suffering from feelings of depression for example, could subsequently lead to less perceived resources such as social support or feeling protected by local authorities.

Future prospective, multi-perspective and longitudinal studies with an even larger number of participants and assessed time points addressing a causal attempt in terms of the uncovered complex interplay between variables of emotional and supportive human relations, mental burden and quality of life of HCW would be advantageous.

## Supplementary Information

**Additional file 1. Table S1:** Correlation analyses of sociodemographic, work- and COVID-19 related control variables with outcome variables in patients working in the outpatient care (POC) at T2.

**Additional file 2. Table S2: **Description and comparability of the subsamples PIC at T1 and T2 and POC at T1 und T2.

## Data Availability

The datasets used and/or analysed during the current study available from the corresponding author on reasonable request.
