# Supplementary material for: Mental distress of physicians in the outpatient care throughout the COVID-19 pandemic: emotional and supportive human relations matter – Cross-sectional results of the VOICE-study
Source: BMC Health Serv Res. 2023 May 12;23:481. doi: 10.1186/s12913-023-09361-3 (PMC10180613; doi:10.1186/s12913-023-09361-3)
Supplement: Supplementary file 1 — Additional file 1. Table S1: Correlation analyses of sociodemographic, work- and COVID-19 related control variables with outcome variables in patients working in the outpatient care (POC) at T2. [file 12913_2023_9361_MOESM1_ESM.docx]

**Supplementary material**

*Table S1:* Correlation analyses of sociodemographic, work- and COVID-19 related control variables with outcome variables in patients working in the outpatient care (POC) at T2.

|  | **Current Burden**  **T2**  r  p  n | **PHQ-2 --**  **T2**  r  p  n | **GAD-2 --**  **T2**  r  p  **n** | **QoL --**  **T2**  r  p  **n** |
| --- | --- | --- | --- | --- |
| **Gender** | -.036  .522  312 | -.065  .268  291 | .027  .642  291 | -.002  .973  289 |
| **Age-group** | -.030  .592  312 | -.022  .704  291 | -.092  .119  291 | .040  .502  289 |
| **Care for relatives** | -.006  .912  312 | -.055  .348  291 | .016  .789  291 | .103  .081  289 |
| **Having children** | .064  .257  312 | .155*  .050  291 | .052  .380  291 | -.143*  .015  289 |
| **Work**  **experience** | -.061  .281  312 | -.008  .888  291 | -.027  .643  291 | -.052  .380  289 |
| **Working**  **Fulltime/Parttime** | -.239**  <.001  312 | -.132*  .024  291 | -.121*  .039  291 | .059  .315  289 |
| **Work in Homeoffice** | -.029  .607  312 | .048  .416  291 | .042  .478  291 | .027  .646  289 |
| **Change of Department** | -.044  .435  310 | -.048  .415  291 | .014  .810  291 | .055  .351  289 |
| **Contact COVID** | .219**  <.001  310 | .091  .121  291 | .160**  .006  291 | -.056  .340  289 |
| **Being at risk**  No-Yes | -.056  .323  310 | .019  .745  291 | -.058  .324  291 | .009  .887  289 |
| **Infection** No-Yes | -.083  .147  310 | -.048  .418  291 | -.085  .146  291 | .036  .546  289 |
| * significance at p ≤ .05, ** p ≤ .01 | | | | |
