# Supplementary material for: Mental distress of physicians in the outpatient care throughout the COVID-19 pandemic: emotional and supportive human relations matter – Cross-sectional results of the VOICE-study
Source: BMC Health Serv Res. 2023 May 12;23:481. doi: 10.1186/s12913-023-09361-3 (PMC10180613; doi:10.1186/s12913-023-09361-3)
Supplement: Supplementary file 2 — Additional file 2. Table S2: Description and comparability of the subsamples PIC at T1 and T2 and POC at T1 und T2. [file 12913_2023_9361_MOESM2_ESM.docx]

**Supplementary material**

*Table S2:* Description and comparability of the subsamples PIC at T1 and T2 and POC at T1 und T2.

| **Variables of interest/**  **Control-variables** | **Physicians Outpatient Care (POC)**  T1+T2  N (%)  848 (100) | **POC** (T1)  n (%)  536 (100) | **POC** (T2)  n (%)  312 (100) | POC T1/T2  p  **Cramer`s V** | **Physicians inpatient care (PIC)**  **Total**  N (%) =  458 (100) | **PIC** (T1)  n(%)  262(100) | **PIC** (T2)  n(%)=196(%) | PIC  T1/T2  p  **Cramer`s V** |
| --- | --- | --- | --- | --- | --- | --- | --- | --- |
| **Sociodemographic** | | | | | | | | |
| **Gender**  Male  Female  Diverse | 342 (40.3)  505 (59.6)  1 (0.1) | 216 (40.3)  320 (59.7)  0 (0%) | 126 (40.4)  185 (59.3)  1 (0.3) | .422  .045 | 330 (44.6)  408 (55.3)  1 (0.1) | 129 (49.2)  133 (50.8) | 101 (51.5)  95 (48.5) | .627  .023 |
| **Age-group in years**  18-30  31-40  41-50  51-60  61-70  >70 | 7 (0.8)  70 (8.3)  194 (22.9)  370 (43.6)  183 (21.6)  24 (2.8) | 6 (1.1)  51 (9.5)  137 (25.6)  219 (40.9)  112 (20.9)  11 (2.1) | 1 (0.3)  19 (6.1)  57 (18.3)  151 (48.4)  71 (22.8)  13 (4.2) | .011  .133 | 6 (0.8)  63 (8.5)  168 (22.8)  323 (43.8)  162 (22.0)  16 (2.2) | 3 (1.1)  25 (9.5)  67 (25.6)  108 (41.2)  55 (21.0)  4 (1.5) | 1 (0.5)  12 (6.1)  37 (18.9)  98 (50.0)  46 (23.5)  2 (1.0) | .235  .122 |
| **Care for relatives**  Yes, own household  Yes, not own household  No | 38 (4.5)  155 (18.3)  655 (77.2) | 23 (4.3)  86 (16.0)  427 (79.7) | 15 (4.8)  69 (22.1)  228 (73.1) | .074  .078 | 29 (3.9)  131 (17.8)  578 (78.3) | 8 (3.1)  33 (12.6)  221 (84.4) | 6 (3.1)  35 (17.9)  155 (79.1) | .291  .073 |
| **Children**  Yes own household  Yes not own household  No | 394 (46.5)  294 (34.7)  160 (18.9) | 261 (48.7)  168 (31.3)  107 (20.0) | 133 (42.6)  126 (40.4)  53 (17.0) | .028  .092 | 345 (46.7)  217 (29.4)  176 (23.8) | 127 (48.5)  73 (27.9)  62 (23.7) | 98 (50.0)  57 (29.1)  41 (20.9) | .784  .033 |
| **Work-related** | | | | | | | | |
| **Professional experience**  <3 years  3-6 years  >6 years  Not direct patient care | 17 (2.0)  21 (2.5)  800 (94.3)  10 (1.2) | 14 (2.6)  14 (2.6)  499 (93.1)  9 (1.7) | 3 (1.0)  7 (2.2)  301 (96.5)  1 (0.3) | .106  .085 | 9 (1.2)  29 (3.9)  615 (83.3)  85 (11.5) | 6 (2.3)  9 (3.4)  233 (88.9)  14 (5.3) | 4 (2.0)  7 (3.6)  174 (88.8)  11 (5.6) | .997  .011 |
| **Full-time/Part-time**  Full-time  Part-time | 683 (80.5)  165 (19.5) | 428 (79.9)  108 (20.1) | 255 (81.7)  57 (18.3) | .505  .023 | 499 (67.6)  239 (32.4) | 191 (72.9)  71 (27.1) | 141 (71.9)  55 (28.1) | .820  .011 |
| **Homeoffice**  Yes completely  In parts  No | 19 (2.2)  170 (20.0)  659 (77.7) | 18 (3.4)  119 (22.2)  399 (74.4) | 1 (0.3)  51 (16.3)  260 (83.3) | .001*  .126 | 10 (1.4)  167 (22.6)  561 (76.0) | 2 (0.8)  46 (17.6)  214 (81.7) | 0 (0.0)  28 (14.3)  168 (85.7) | .293  .073 |
| **Change of department**  Yes  No | 83 (9.8)  759(89.5) | 66 (12.4)  466 (87.4) | 17 (5.5)  293 (94.5) | .004*  .115 | 93 (12.6)  643 (87.4) | 43 (16.5)  217 (83.5) | 21 (10.8)  173 (89.2) | .084  .081 |
| **COVID-19 related** | | | | | | | | |
| **Contact w. COVID-19** (infected patients and/or contaminated material)  Yes  No | 496 (58.5)  347 (40.9) | 259 (48.6)  274 (51.4) | 237 (76.5)  73 (23.5) | <.001*  .273 | 359 (48.7)  378 (51.3) | 151 (58.1)  109 (41.9) | 116 (60.1)  77 (39.9) | .151  .067 |
| **Being at risk**  Yes  No | 381 (44.9)  461 (54.4) | 225 (42.3)  307 (57.7) | 156 (50.3)  154 (49.7) | .024  .078 | 318 (43.2)  418 (56.8) | 103 (39.6)  157 (60.4) | 92 (47.4)  102 (52.6) | .096  .078 |
| **Infection**  Yes  No  Don´t know | 12 (1.4)  590 (69.6)  239 (28.2) | 4 (.9)  345 (64.8)  182 (34.2) | 8 (2.6)  245 (79.0)  57 (18.4) | <.001*  .183 | 16 (2.2)  720 (97.8) | 6 (2.3)  152 (58.5)  102 (39.2) | 8 (4.1)  151 (78.2)  34 (17.6) | <.001*  .235 |
| *significance after Bonferroni-correction (p ≤ .05/11)=.005 | | | | | | | | |
